# Hypoxic environment may enhance migration/penetration of endocrine resistant MCF7- derived breast cancer cells through monolayers of other non-invasive cancer cells *in vitro*

**DOI:** 10.1038/s41598-020-58055-x

**Published:** 2020-01-24

**Authors:** Nora H. Barrak, Maitham A. Khajah, Yunus A. Luqmani

**Affiliations:** Faculty of Pharmacy, Kuwait University, Safat, 13110 Kuwait

**Keywords:** Cancer, Cell biology, Molecular biology, Oncology

## Abstract

The response of cancer cells to hypoxic conditions found within the interior of a tumor mass is mediated through the hypoxia inducible factor (HIF) cascade and is thought to promote metastasis. However, given their distant proximity from blood vessels as compared to normoxic cells at the vascularised tumor periphery, it is uncertain if these cells can migrate through the tumor mass to gain access. Hypoxia was simulated by exposure to cobalt chloride or deferoxamine in normal (MCF10A) and cancerous [estrogen receptor (ER)−ve (pII), and ER +ve (YS1.2/ EII)] cells. In this report, HIF1α expression and localization was measured using western blotting, ELISA, and immunofluorescence, cell proliferation by MTT assay, motility and invasion by wound healing, live cell imaging, matrigel and co-culture in chambered slides. We found that the expression and nuclear translocation of HIF1α was significantly elevated by hypoxia, which inhibited cell proliferation, but significantly increased motility of pII cells and their penetration into and through a dense layer of adjacent EII cells, as well as their selective emergence out of a co-culture. These data suggest that endocrine resistant pII cancer cells, having undergone epithelial to mesenchymal transition are able to penetrate through other cell layers, with possible enhancement in response to hypoxia.

## Introduction

Hypoxia is a state of low oxygen level, and represents a pernicious effect of diseases such as cardiovascular diseases, diabetes, dementia and cancer. It is variably observed in most if not all solid tumors, depending on the type, size and stage^1^ and develops as a result of expansion of tumor tissue due to increased proliferation of cancer cells with reduced oxygen supply especially in the core where there is considerable distance from the tumour-induced vasculature^2^. This condition is correlated with poor prognosis as well as poor outcome of anti-cancer therapies^3–5^.

In the breast, hypoxia has been shown to induce genome-wide changes in DNA hydroxy-methylation that leads to the acquisition of breast tumor-initiating cell properties with up-regulation of tumor necrosis factor and activation of the p38-MAPK effector pathway^6^. Acute and chronic states of hypoxia can lead to variable outcomes in cancer cells^7^. Under conditions of acute hypoxia they adapt by decreasing reliance upon oxidative metabolism for energy generation, while increasing autophagy, reactive oxygen species (ROS) generation and metastasis, leading to tumor survival and progression^8–12^. Chronic hypoxia for a few hours to several weeks leads to high frequency of DNA breaks and accumulation of replication errors leading to genetic instability and mutagenesis^13–15^. Hypoxia also appears to be a driver of alternative splicing events in a large number of genes involved in multiple processes of tumorigenic development^16^.

The hypoxia-inducible factor (HIF)^17^, is considered to be the major downstream transcription factor activated in both hypoxic and non-hypoxic conditions by stimuli such as nitric oxide (NO), ROS, cytokines, lipopolysaccharide, G-protein coupled receptors and toll-like receptors^18–21^. In the presence of oxygen, HIF-1α subunits are hydroxylated by oxygen-dependent enzymes such as prolulhydroxylases and factor inhibiting HIF-1^22^ leading to poly-ubiquitination and proteasomal degradation of the α subunits^23^. Under hypoxic conditions, these oxygen-sensor enzymes lose their activity resulting in HIF-1α protein stabilization, accumulation and translocation to the nucleus to dimerize with the constitutively expressed HIF-1β subunits to bind to target hypoxia-responsive elements and induce expression of genes involved in cell anaerobic metabolism, survival, metastasis, angiogenesis and drug resistance. These include VEGF, SDF-1, Ang-II, MMPs 2 and 9, BNIP-3, p53, E-cadherin, CXCR4, LOX, CAIX, GLUT-1, and GSK^22,24–30^. A recent study indicated that the TRAF6-H2AX-γH2AX- axis mediates HIF1α enrichment in the nucleus of cancer cells leading to its stabilization and activation to enhance tumorigenesis, glycolysis and metastasis^31^. HIF-1-α was shown to be expressed in the majority of cancer biopsies at the leading edge of invading tumors^32^ and correlated with increased risk of metastasis and poor prognosis^33–43^.

Hypoxia can be stimulated *in vitro* by incubation in low oxygen environment using a specialized chamber, or by incubation with chemical agents. Exposure to cobalt chloride (CoCl_2_) (which *in vivo* is a chelating agent replacing Fe^2+^ in hemoglobin, impairing the cell’s reception of oxygen^44,45^) was shown to induce HIF-1α expression in PC-2 human pancreatic cancer cells^46^. Deferoxamine (DFO), a bacterial sidephore that chelates iron and inhibits iron-dependent prolyl hydroxylases thus preventing the degradation of HIF isoforms in normoxic conditions^47–49^ has also been used to induce a state of hypoxia *in vitro*. Kilic and colleagues recently showed increased release of extracellular vesicles from MCF-7 breast cancer cells, due to CoCl_2_ induced hypoxia^50^. These are increasingly implicated in cancer progression through inter-cellular communication and transfer of mediators.

It seems to be generally held that hypoxic environment modulates various effector functions of cancer cells such as induction of epithelial to mesenchymal transition (EMT)^51–53^, and enhancement of cell proliferation^45^ and invasion^54^ which promotes tumour metastasis. This has led to the popular belief that metastasis is promoted by an acidic extracellular environment, which is expected to be found around anaerobically metabolizing cells producing and secreting excessive amounts of lactate (and by co-transport, H+), i.e. the hypoxic cells. It has also been suggested that there may be a ‘metabolic symbiosis’ between hypoxic cells producing lactate and oxygenated cells utilizing it as a substrate to feed into their oxidative cycle. However, the obvious but apparently un-addressed anomaly here is two-fold. Firstly, the hypoxic cells are buried within the core of the tumour and would have to penetrate through a dense meshwork of more oxygenated cancer and normal cells to reach the tumour periphery to contact blood vessels. Secondly, it is the cells at the neo-vascularized normoxic surface of the tumour mass that are actively proliferating, and therefore logically may be expected to have greater opportunity to metastasise. So we are faced with this intriguing question – which cells in the tumour mass actually metastasise? Clearly this issue will not be easy to resolve and longer term may be best addressed by video imaging of tumours *in vivo* when this becomes possible.

In the current report it is intended to look at this question in the limited setting of an *in vitro* model that could provide some preliminary indications to address the questions posed above. Using the weakly-invasive estrogen receptor (ER) +ve MCF7 parental cells and the highly invasive ER silenced pII cells, we proposed to firstly examine their relative proliferative, motile and invasive abilities under normoxic/hypoxic conditions, comparing these also with normal MCF10A breast epithelial cells. Then, to try to simulate a tumor mass by mixing the different cells to determine whether they can penetrate through layers of each other before/after pre-treatment with HIF1α inducing agents to simulate the conditions of hypoxia.

## Materials and Methods

### Cell lines

MCF10A (used in this study as a normal non-malignant breast cell line) was obtained from Dr E Saunderson St Bartholomews Hospital, London. MCF7 (estrogen receptor ER +ve breast cancer cells) were originally obtained from the ATCC (American Type Culture Collection, VA, USA). pII (ER −ve) and EII (ER +ve) are stable shRNA transfected cloned lines derived from the MCF7 line^55^. pII is ER-silenced while EII is a control line containing the shRNA carrying plasmid without ER down-regulation and constitutively expressing green fluorescent protein (GFP) as a marker. YS1.2 is MCF7 transfected with ER-directed shRNA but also failed to down-regulate ER and remained ER +ve^56^.

MCF10A cells were cultured in DMEM F12 medium supplemented with 5% horse serum, 1x Pen/Strep, 20 ng/mL EGF, 0.5 µg/mL hydrocortisone, 100 ng/mL cholera toxin and 10 µg/mL insulin. All other cell lines were routinely maintained at 37 °C in a humidified atmosphere of 5% CO_2_ in Dulbecco’s modified eagle’s medium (Advanced DMEM), supplemented with 5% fetal bovine serum (FBS), 600 mg/mL L-glutamine, 100 U/Ml penicillin, 100 mg/mL streptomycin and 6 mL/500 mL 100 x non-essential amino acids. Cells were routinely grown in monolayer in 25 or 75 cm^2^ tissue culture flasks inside an incubator maintained at 37 °C with 5% CO_2_ atmosphere at 95% humidity.

Cell cultures were periodically treated with mycoplasma removal agent from Biorad (USA) and tested with detection kits from Invivogen (USA) and DAPI nuclear staining to ensure they remained free of mycoplasma.

### Cell labeling

Qtracker 625 cell labeling kit (ThermoFisher Scientific, USA) was used to label pII cells (red dye) to monitor their motility. This was performed by mixing 1 µl each of solution A and B for 5 min followed by addition of 200 µl DMEM and mixing with 1 × 10^6^ pII cells prior to incubation at 37 °C in 5% CO_2_ for 1 h. Following this incubation, the media was discarded and replaced with fresh media. The dye has an excitation of 405–585 nm and emission of 625 nm.

### Western blotting

Cells were cultured in 6 well plates with complete DMEM to 80–90% confluence, and the medium was subsequently aspirated off and cell monolayers harvested by scraping and re-suspension into 300 μl of lysis buffer containing 50 mM HEPES, 50 mM NaCl, 5 mM EDTA 1% Triton X, 100 µg/ml PMSF, 10 µg/ml aprotinin, and 10 µg/ml leupeptin. Protein concentration was determined by the Bradford assay using BSA as standard, and 8 μg protein lysate was mixed with an equal volume of 2 x SDS and heated at 90 °C for 10 min. Samples were loaded onto a 10% SDS-polyacrylamide gel and electrophoresed at 150 V for 1 h. Proteins were transferred to a nitrocellulose membrane and blocked with 2% BSA for 1 h before being incubated overnight at 4 °C with HIF1α or actin (loading control) antibody (Cell Signaling, USA) (1:1000 dilution) prepared in 2% BSA. The membrane was washed and incubated with anti-HRP-conjugated secondary antibody (Cell Signaling, USA) (1:1000 dilution) for 1 h, developed with Super Signal ECL and visualized with Kodak X-ray film.

### Immunofluorescence

Cells were seeded at approximately 5000 cells/well in an 8-chambered slide containing 200 µl/well DMEM media (CO_2_ independent media) and allowed to settle for 48 h. The media was then removed and replaced with media containing either Cobalt (II) chloride (CoCl_2_) or Deferoxamine mesylate salt (DFO) (Sigma-Aldrich, USA) at 100 µM and left for 30 min, 1 h, 4 h, 24 h and 48 h at which times cells were fixed by the addition of 3.7% formaldehyde for 10 min and washed with ice-cold PBS. Cells were treated with 1% BSA and then incubated with HIF1α antibody (1:80 dilution) overnight at 4 °C. Unbound primary antibody was aspirated, cells washed with ice-cold PBS and incubated with anti-rabbit secondary antibody (1:250 dilution). Excess unbound secondary antibody was aspirated off, cells were washed with ice-cold PBS, fixed and stained with phalloidin (Thermo-fisher, USA) (1:125 dilution) to stain for F-actin and DAPI to stain the nucleus. After removal of the silicone scaffolding and mounting, cells were examined under a confocal microscope (Carl Zeiss, Germany) at an excitation wavelength of 450 nm and images analyzed and processed using a ZEISS software package.

### MTT assay

Cells were seeded into 24-well culture plates and allowed to grow to 30–35% confluency. The medium was removed and replaced with medium containing 100 µM CoCl_2_ or DFO or vehicle, and cell quantity determined either immediately (day zero) or after 1 day and 4 days of growth. For the measurement, medium was removed and replaced with 500 µl of MTT reagent (Sigma-Aldrich, USA) (0.5 mg/ml) and left at 37 °C for 2 h; MTT solution was removed and 200 µl of acidic isopropanol added to dissolve the blue formazan crystals. Plates were scanned at 595 and 650 nm using a MULTISKAN SPECTRUM spectrophotometer.

### Motility assay

Cells were grown to 90% confluency in 12 well plates, and treated with vehicle (dH_2_O), or various concentrations (25–100 µM) of CoCl_2_ or DFO. A fine scratch was made using a sterile yellow 200 µl Eppendorf pipette tip through the center of the YS1.2 cell monolayer and 1000 µl tip for pII and MCF10A cell monolayers. The width of the scratch was recorded under phase contrast light microscopy immediately (0 h) and after 24 h of incubation to determine the extent of wound closure.

### Live cell imaging

Relative movements of the different cell lines were studied using an Ibidi µ chamber plate (Ibidi, Germany) which allows segregated cultivation of cells. Non-invasive EII cells, which have intrinsic green fluorescence due to expression of GFP, and the highly invasive pII cells (previously labeled with the Qtracker red dye) were grown in adjacent chambers of an Ibidi plate (1 × 10^4^ cells in 100 µl of DMEM media) at 37 °C, 5% CO_2_ overnight to attach. On the following day, the spacer between the cells was removed and 2 ml of CO_2_ independent culture media (to maintain pH at 7.4) was added and the slide placed inside an imaging chamber (Cell Observer HS, ZEISS) heated to 37 °C with an airstream. Live cell microscopy with time-lapse photography, was used to continuously monitor the migration of cells (pII and EII) into their opposite compartment. For this, the plate was positioned to enable photography of both cells on the edges and an empty space in the middle and images (at 10 x magnification) were taken every 10 min. The AxioVision software (ZEISS) was used to combine all the pictures to generate a video.

### Cell invasion through a single layer of basement membrane extract

The upper inset chambers of a Cultrex assay dish were coated with 100 µl of 1x basement membrane extract (BME) solution and incubated overnight at 37 °C. Meanwhile, cells (treated with vehicle, or 100 µM CoCl_2_ or DFO) were serum starved overnight at 37 °C and 5% CO_2_. On the following day, cells were harvested and re-suspended in serum free media and 100 µl containing 10^5^ cells were added into the upper chamber. The lower chamber was loaded with 500 µl of serum free media (negative control) or media containing 40% FBS as a chemo-attractant. Cells were incubated at 37 °C, 5% CO_2_ and allowed to invade from the top to the bottom chamber. After 48 h, the media was aspirated from the upper chamber and the bottom washed with 1x cell washing buffer. Calcein-AM/cell dissociation solution was added to the bottom chamber and left for 1 h at 37 °C, 5% CO_2_. Cells present in the bottom chamber were quantitated by their ability to internalize Calcein-AM, with intracellular esterases cleaving the acetomethylester (AM) moiety generating free fluorescent calcein which could then be determined by recording the fluorescence emission using a microplate reader with a filter set of Excitation/Emission = 485/535 nm (CULTREX, 2008).

### Cell invasion through a two-layer system composed of cells and BME

The Cultrex inset chamber was coated with 1x BME and left to set overnight as previously described. On the next day, serum starved EII cells were re-suspended in serum free media and 100 µl of cells (10^5^) were added into the upper chamber and allowed to settle at 37 °C overnight. Then, 100 µl of pII cells (10^5^) that had been 24 h serum starved either in the absence (control) or presence of 100 µM CoCl_2_ overnight at 37 °C and 5% CO_2_ and re-suspended in serum free media were added to form a layer on top of the EII cells. The plate was then left overnight at 37 °C in 5% CO_2_ and procedure followed as already described above.

### Under-agarose migration assay

Ultra-pure agarose (Invitrogen, USA) (0.18 g) was dissolved by boiling in 10 ml dH_2_O and allowed to cool below 40 °C, then supplemented with complete DMEM to make a final 0.5% solution and allowed to solidify in individual wells of a 6 well plate at room temperature. Once set, 1–2 sample chambers (3.5 mm in diameter) were created in the gel, 2.5 mm apart in a horizontal line, by insertion of a metallic mold as described previously^57^. Suspensions of pII and YS1.2 cells (4 × 10^4^) in DMEM containing 5% FBS, that had previously been exposed for 24 h to CoCl_2_ or DFO (100 µM) or to vehicle only were loaded into the formed chambers. Plates were incubated at 37 °C in 5% CO_2_ humidified atmosphere. After 24 h, cells that had penetrated the agarose were manually counted by visual microscopic examination as previously described^57^.

### Ibidi invasion assay

To monitor the migration/invasion of labeled pII cells towards EII cells, 1 × 10^4^ cells suspended in 100 µl of DMEM were grown in separate compartments of an Ibidi chamber plate. The plate was incubated at 37 °C, 5% CO_2_ overnight. On the next day, the spacer between the cells was carefully removed and replaced with 2 ml of DMEM containing CoCl_2_ or DFO (100 µM) or vehicle (control). The number of pII cells migrating/invading through EII cells in all the conditions was recorded under phase contrast and fluorescence light microscopy immediately after removing the spacer and at various intervals thereafter (24–96 h).

### Ibidi invasion assay with co-cultured cells

To monitor the migration of labeled pII cells out of a co-culture with EII cells, the two cell cultures (1 × 10^4^) suspended in 100 µl of DMEM were mixed together in different proportions (EII:pII; 1:1 and 4:1) in an Ibidi chamber plate. The plate was incubated at 37 °C, 5% CO_2_ overnight. On the next day, the spacer was removed and medium replaced with 2 ml DMEM containing CoCl2 (100 µM) or vehicle. The number of pII cells moving/invading out of the mixture was recorded as previously described.

### Statistical analysis

Difference between mean values of tested groups was analyzed by the student’s t-test or one-way ANOVA followed by Bonferroni post-hoc test using GraphPad Prisim 5. P < 0.05 was considered statistically significant.

## Results

### Effect of hypoxic simulation with CoCl_2_ and DFO on HIF-1α expression in normal and cancerous breast epithelial cells

**Figure 1:**
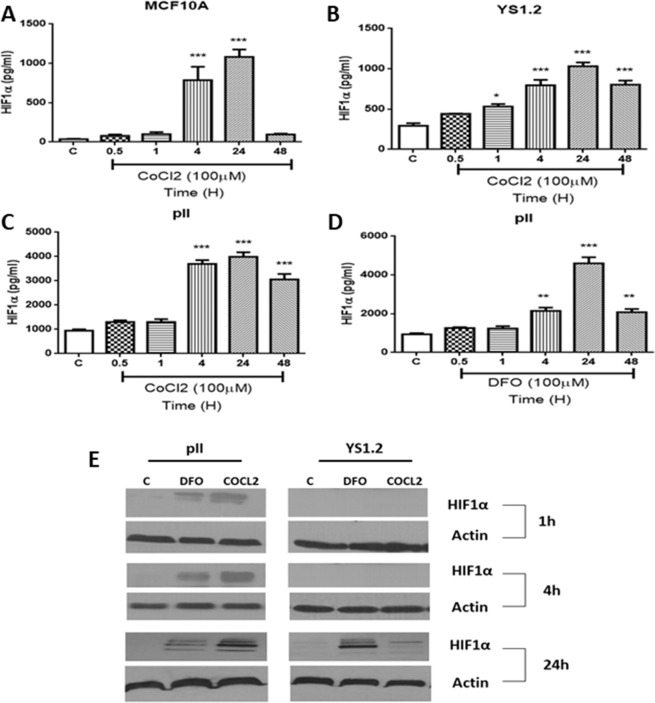
Effect of CoCl_2_ and DFO treatment on HIF-1α expression. (**A**), normal breast epithelial cells MCF10A (**B**), estrogen receptor (ER) positive YS1.2 and (**C,D**), ER-negative pII breast cancer cells either untreated (control, open bars) or treated with CoCl_2_ or DFO (100 µM, hatched bars). HIF-1α was measured in the cell lysate by ELISA at times indicated as described in Methods. Histobars represent the mean ± SEM of 3 independent determinations. Asterisks denote significant difference from the control, with (*)p > 0.05, (**)p = 0.01, and (***)p = 0.001. Panel (E) Western blot analysis of HIF-1α in breast cancer cells, which were either untreated (control) or treated with CoCl_2_ or DFO (100 µM) and HIF-1α determined at the times indicated. Protein lysates were electrophoresed in 10% SDS-polyacrylamide gel and blotted onto nitrocellulose membrane which was then cut into narrow strips (in the region of expected bands) and individually probed with antisera to HIF1α (bands were detected at a molecular weight of 120 kDa which corresponds to the expected HIF1α size), or to β-actin, used as loading control.

The optimal concentration for induction of HIF-1α was found to be 100 µM for both CoCl_2_ and DFO and this was used for subsequent experiments. Using an ELISA-based technique, HIF-1α protein levels were determined in untreated (control) cells and at various times (0.5–48 h) after addition of CoCl_2_ or DFO. As shown in Fig. 1A–C, the baseline level of HIF1α in untreated control MCF10A cells (40 ± 4.34 pg/ml), was much lower than that in both ER +ve (300 ± 32.03 pg/ml) or ER−ve (1000 ± 59.21 pg/ml) breast cancer cells. In all cases, HIF1α levels reached a maximum by 24 h of treatment and declined thereafter. The CoCl_2_ induced level of HIF1α observed in the ER−ve pII cells after 24 h was of four-fold greater magnitude than that seen in MCF10-A and YS1.2 cells. But interestingly the relative increase in HIF1α from the untreated control was approximately 26–30 fold in MCF10A cells and only 3–4 fold in the cancer cells. The effect of DFO was also tested on pII cells and was found to be similar to that of CoCl_2_ (Fig. 1D). Western blot analysis was also performed to confirm the data obtained using ELISA (Fig. 1E).

### Effect of hypoxia simulation on HIF-1α localization

**Figure 2:**
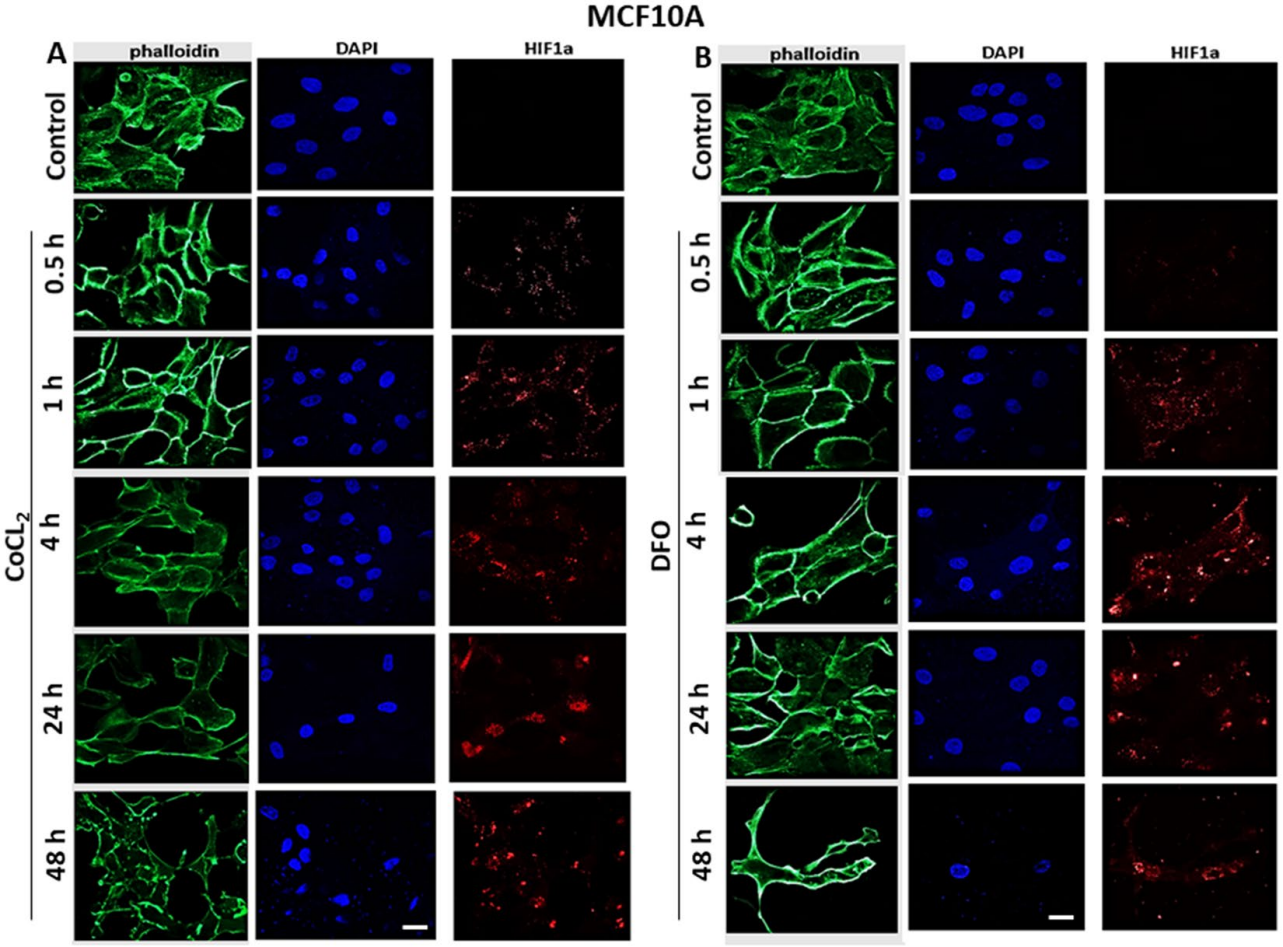
Effect of CoCl_2_ or DFO treatment on HIF-1α localization in normal breast epithelial cells. MCF10A cells were seeded in 8-chamber Ibidi slides and allowed to grow for 48 h at 37 °C/ 5% CO_2_. Cells were either untreated (control) or treated with (**A**) CoCl_2_ or (**B**) DFO (100 µM) for times indicated. Cells were then fixed and stained with HIF1α antibody (red), phalloidin (green, to stain F-actin cytoskeleton), and DAPI (blue, to stain the nucleus), 20x magnification. Scale bar represents 20 µm.

**Figure 3:**
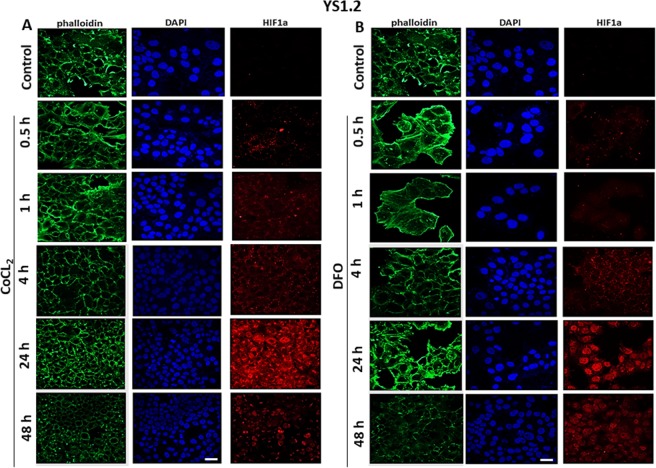
Effect of CoCl_2_ or DFO treatment on HIF-1α localization in ER +ve breast cancer cells. YS1.2 cells were seeded in 8-chamber Ibidi slides and allowed to grow for 48 h at 37 °C/ 5% CO_2_. Cells were either untreated (control) or treated with (**A**) CoCl_2_ or (**B**) DFO (100 µM) for times indicated. Cells were then fixed and stained with HIF1α antibody (red), phalloidin (green, to stain F-actin cytoskeleton), and DAPI (blue, to stain the nucleus), 20x magnification. Scale bar represents 20 µm.

**Figure 4:**
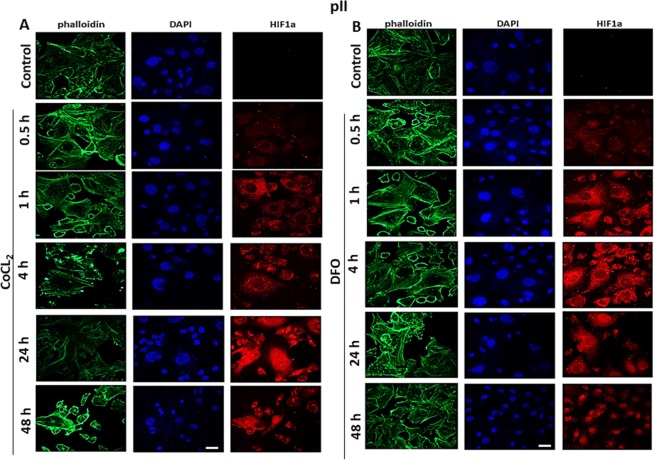
Effect of CoCl_2_ or DFO treatment on HIF-1α localization in ER−ve breast cancer cells. pII cells were seeded in 8-chamber Ibid slides and allowed to grow for 48 h at 37 °C/ 5% CO_2_. Cells were either untreated (control) or treated with (**A**) CoCl_2_ or (**B**) DFO (100 µM) for times indicated. Cells were then fixed and stained with HIF1α antibody (red), phalloidin (green, to stain F-actin cytoskeleton), and DAPI (blue, to stain the nucleus), 20x magnification. Scale bar represents 20 µm.

The distribution pattern of HIF1α was determined by immunofluorescence in response to CoCl_2_ or DFO treatment (100 µM) at 30 min to 48 h. Cells were fixed and labeled with HIF1α antibody, phalloidin (to visualize F-actin) and DAPI (nuclear stain). Treatment of MCF10A cells with either CoCl_2_ (Fig. 2A) or DFO (Fig. 2B) showed peri-nuclear expression of HIF-1 α at 4–48 h with no detectable expression at earlier times (0.5–1 h). In regard to YS1.2 cells (Fig. 3), peri-nuclear HIF-1α expression was induced in response to CoCl_2_ (A) or DFO (B) treatment at earlier times (0.5–4 h) while nuclear expression peaked at 24 h. In regard to pII cells (Fig. 4), peri-nuclear and cytoplasmic expression of HIF-1α was induced at earlier times (0.5–4 h) in response to CoCl_2_ (A) or DFO (B) treatment but at 24–48 h of stimulation HIF-1α was detected in the nucleus. Due to its poor immunoreactivity (possibly due to low expression) HIF1α staining in all the figures (Figs. 2–4) was digitally enhanced in order to assess its cellular localization.

### Effect of hypoxic simulation on cell proliferation

**Figure 5:**
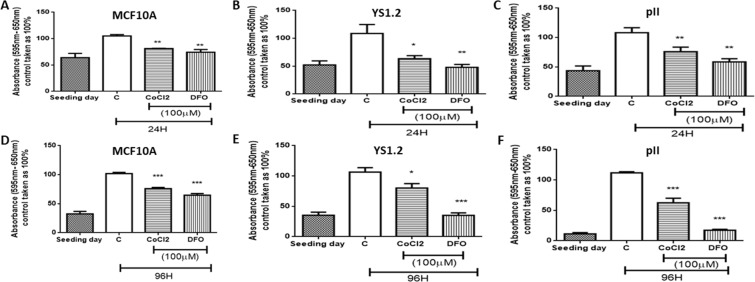
Effect of CoCl_2_ or DFO treatment on cell proliferation. Cells, as indicated, were seeded at day 1 (hatched bar) were either untreated (C) or treated with CoCl_2_ or DFO (100 µM) and assayed by MTT after 1 day (**A–C**) or 4 days (**D–F**). Histobars represent the mean ± SEM of 6 independent determinations. Asterisks denote significant difference from the control, with (*)p > 0.05, (**)p = 0.01, and (***)p = 0.001.

The effect of CoCl_2_ or DFO (100 µM) treatment on cell proliferation was assessed using the MTT assay. As shown in Fig. 5, treatment with CoCl_2_ or DFO decreased proliferation of both normal (MCF10A) as well as the cancerous cells (YS1.2 and pII). This inhibition was seen at 24 h (A-C) and maintained at 96 h (D-F). The effect was more profound in the cancerous cells particularly at the shorter time and was also more pronounced with DFO. As this was somewhat unexpected, and to ensure that the results were not due to any impairment in mitochondrial function (at least in the enzymes involved in the MTT conversion) we also performed a proliferation assay with manual cell counting using a haemocytometer and found similar results (data not shown) to using the MTT assay.

### Effect of hypoxic simulation on breast cancer cell motility

**Figure 6:**
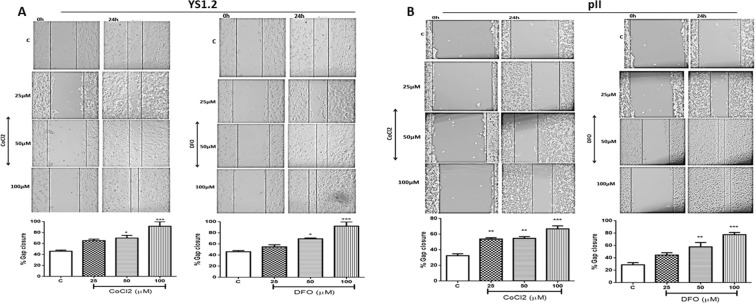
Effect of CoCl_2_ or DFO treatment on breast cancer cell motility. A scratch was made through a confluent layer of (**A**) YS1.2 or (**B**) pII cells, using a p20 (for YS1.2) or p1000 (for pII) pipette tip, and the gap measured at 0 time and after 24 h of DFO or CoCl_2_ treatment at the concentrations indicated, as described in Methods. The % wound closure was calculated as the width of the empty space after 24 h divided by the width at 0-time x 100 subtracted from 100. Histobars represent the mean ± SEM of 9 independent determinations. Asterisks denote significant difference from the control, with (*)p > 0.05, (**)p = 0.01, and (***)p = 0.001.

Wound healing (scratch) assay was used to determine the effect of hypoxic simulation on the motility of ER +ve and ER −ve breast cancer cells. As illustrated in Fig. 6, both CoCl_2_ and DFO treatment significantly increased YS1.2 (A) and pII (B) cell motility with maximal effect at 100 µM. It should be noted that the scratch in YS1.2 was made with a yellow tip as compared to a blue tip for the pII scratch creating a much wider gap. Hence the actual movement (as opposed to % closure) of YS1.2 is much less than for pII. We also examined motility of MCF10A cells and surprisingly found that they were highly motile. As their culture medium contains stimulatory additives, we repeated the experiments omitting EGF in one case and in another, replacing the medium (24 h prior) with the DMEM medium used for the cancer cells. The data (Supplementary Fig. 2) showed motility only in the *complete MCF10A medium* with no or little movement in the other two media. The presence of either DFO or CoCl_2_ however, stimulated significant dose dependent motility in all three media.

### Effect of hypoxia on migration and invasion of breast cancer cells

**Figure 7:**
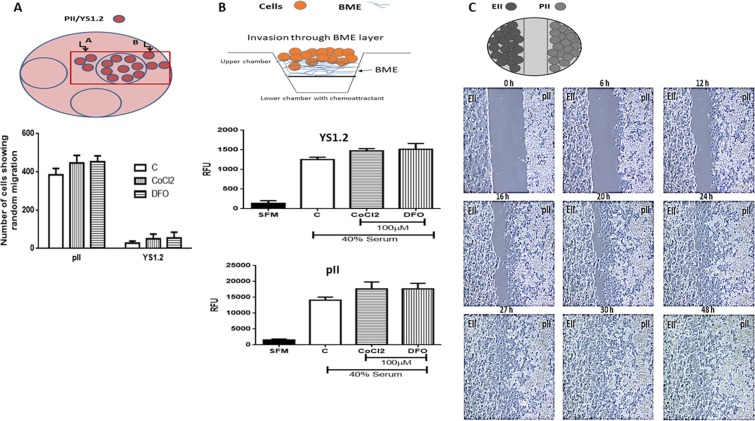
Effect of hypoxic simulation on the migration and invasion of breast cancer cells. Panel A: YS1.2 and pII cells were treated with either vehicle (control), or with or CoCl_2_ or DFO (100 µM) for 24 h. Cells were loaded into wells formed in an agarose layer in 6 well plates. The total number of cells migrating/penetrating into the agarose in both lateral directions were manually counted using an inverted microscope as described in Methods. Histobars represent mean ± SEM of 3 independent determinations. Panel B: YS1.2 and pII cells pre-treated with vehicle (C, open bar), CoCl_2_ or DFO (100 µM, hatched bars) were loaded into the top chamber of a Cultrex dish. Cells invading through the BME layer into the lower chamber containing either serum free media (SFM, solid bar) or media containing 40% serum (used as chemoattractant) were measured as described in Methods and represented here as relative fluorescence units. Histobars represents mean ± SEM of 6 independent determinations. Panel C: Snapshots from time-lapse photography of pII and EII cells cultured for 48 h. Cells were seeded separately into adjacent chambers of an Ibidi chamber slide with a space between them. After 24 h the scaffold was removed leaving a gap between the two monolayers. The time-lapse photography showed that only the pII cells moved towards the EII cell monolayer and over 27 h completely closed the gap. (10x magnification). A schematic diagram explaining each experimental setup is shown on the top of each panel.

The effect of hypoxia on random migration of ER +and ER− breast cancer cells was assessed using the agarose assay. Both YS1.2 and pII cells were pre-treated with either vehicle (control) or CoCl_2_/DFO. As shown in Fig. 7A, pII cells were far more migratory when compared to YS1.2 as previously reported^57^, and CoCl_2_/DFO treatment did not significantly increase the number of cells migrating out of the well into the agarose. The low migratory capacity of YS1.2 cells was not influenced by either drug.

The Cultrex BME cell invasion assay was also used to assess the effect of hypoxic simulation on invasion of YS1.2 and pII cells. In this assay, both YS1.2 and pII cells were pre-treated with vehicle or CoCl_2_/DFO for 24 h and then loaded into the top chamber separated from the bottom chamber containing medium supplemented with 40% serum as a chemo-attractant. Cells invading into the lower chamber were assessed after 24 h. Figure 7B shows that ER −ve cells were 10 fold more invasive (note difference in Y axes) when compared to ER +ve cells; CoCl_2_/DFO treatment did not significantly increase cell invasion of either through the BME layer.

Live cell microscopy was also used to monitor the movement of ER −ve (pII, right side) and ER +ve (EII, GFP-tagged, left side) breast cancer cells co-cultured in an Ibidi chamber plate under normal conditions. As shown in Fig. 7C, within the first 6 h, pII cells had moved towards the EII cell population and completely closed the gap between the two cell populations by 27 h. The time lapse film (supplementary file) shows very clearly that the EII cells did not move towards the pII cells. However, with the optical instruments at our disposal, we were unable to determine whether the pII cells were able to invade further *into* the EII cell monolayer since we could not distinguish between the two cell populations despite that the EII cells were fluorescently labelled with GFP.

**Figure 8:**
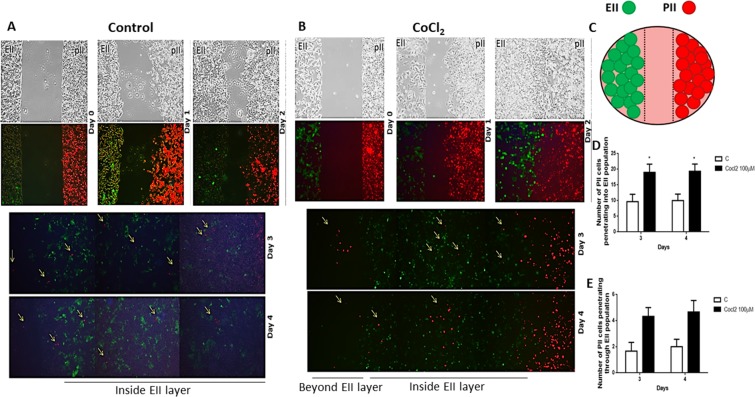
Culture of ER−ve and ER +ve breast cancer cells under normoxic and hypoxic condition. Cells (red; pII, green; EII) were seeded separately (at 80% confluency) into adjacent chambers of an Ibidi chamber slide with a space between them. After 24 h the scaffold was removed leaving a gap between the two monolayers. Cells were cultured under normoxic (panel A, control) or hypoxic conditions (panel B) for 4 days. Snap shots of the cells were taken every 24 h; pII cells moved to close the gap (day 2) and migrate/penetrate into the EII monolayer and later a few cells penetrated through it (days 3–4). Panel C represents a schematic diagram explaining the experimental setup. The number of pII cells penetrating EII monolayer (**D**) or invading through the EII population (**E**) is shown in the histobars; this was manually observed using a fluorescence microscope. Histobars represent 3 independent determinations with (*)p > 0.05.

In order to study this phenomenon further and confirm whether pII cells can invade through a layer of another cancer cell type such as EII cells which are not or much less motile, pII cells were labeled with red dye and grown in an Ibidi chamber plate in a well adjacent to another containing a population of EII cells (green-labeled due to intrinsic GFP expression) and snap shots taken in phase contrast and fluorescence mode at 24 h intervals. In the first experiments we used lower confluency (about 60%) of EII cells (data not shown) and found that many pII cells were present inside the EII layer possibly because there was empty space so we increased EII cells to 80% confluency to test whether pII cells are able to penetrate a more solid barrier of another cell line. As shown in Fig. 8A, two confluent monolayers of pII cells (right panel, red label) and EII cells (left panel, green label) were established and after 3 days, pII cells started to invade the EII population with about 10 cells present inside the EIImonolayer. On the third day of the co-culture, a few pII cells also penetrated beyond the EII layer. No EII cells were observed inside the pII monolayer. Following on from the previous experiment we wished to determine whether hypoxic conditions would alter the observed behaviour of the pII and EII cells (Fig. 8B). Thus we set up the same experiment as described above (Fig. 8A) but in the presence of CoCl_2_ or DFO; snap shots were taken using phase contrast and fluorescence mode every 24 h. As shown in Fig. 8B, after 24 h of exposure under hypoxic conditions (CoCl_2_), pII cells started to move towards the EII monolayer and closed the space between them. On day 2, pII cells began to invade into the EII population, and at day 3 some pII cells penetrated through the EII monolayer. This was also observed with DFO treatment (data not shown). There was a 2-fold increase in the number of pII cells invading the EII monolayer under hypoxic conditions as compared to normoxia (Fig. 8D). Although there was a trend (this did not reach statistical significance) in the number of cells penetrating through the EII layer (Fig. 8E). There was no movement of EII cells towards the pII cell monolayer.

**Figure 9:**
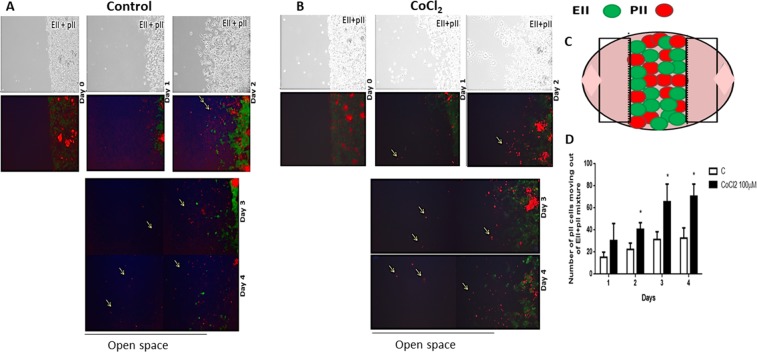
Effect of CoCl_2_ on the migration of mixed population of ER−ve and ER +ve breast cancer cells. Cells (red; pII, green; EII) were seeded together (at 80% confluency) into an Ibidi chamber slide. Cells were cultured under normoxic (**A**) or hypoxic (CoCl_2_ 100 µM) (**B**) condition for 4 days; snapshots of the cells cultured under both conditions taken every 24 h. pII cells migrated/penetrated out of the mixture into the open space starting from day 1 of the co-culture. Panel C represent a schematic diagram explaining the experimental setup. Panel D indicates total number of pII cells (red) moving out of the mixture manually counted every 24 h using a fluorescent microscope. Histobars represent 3 independent determinations. Asterisks denote significant difference from the control, with p < 0.05 (*).

Having observed that pII cells could move into and through a layer of EII cells, we wished to determine how these cells would behave if cultured as a mixed population in both normoxic as well as hypoxic conditions as might be expected within a tumor mass. For this, pII cells were labeled with red dye and grown in an Ibidi chamber plate with EII cells under normoxic condition or in the presence of CoCl_2_. Two experimental settings were performed; a), with an equal ratio of ER +ve and ER −ve cells (data not shown) and b), with ER +ve: ER −ve cells at a ratio of 4:1 (Fig. 9). Under normoxic conditions pII cells were able to migrate out of the mixed population into the surrounding empty space. This was more pronounced when the density of both cell populations was equal compared to when there were fewer pII cells having to migrate through a higher density of EII cells. In the presence of CoCl_2_ the number of pII cells migrating out of the mixed population increased about two-fold (Fig. 9D). Unfortunately, the photographs do not show the proportion of the two cell populations correctly because the red fluorescence overpowers the green; however, in day 2, since the pII cells moved, the green fluorescence is more distinct from the red.

**Figure 10:**
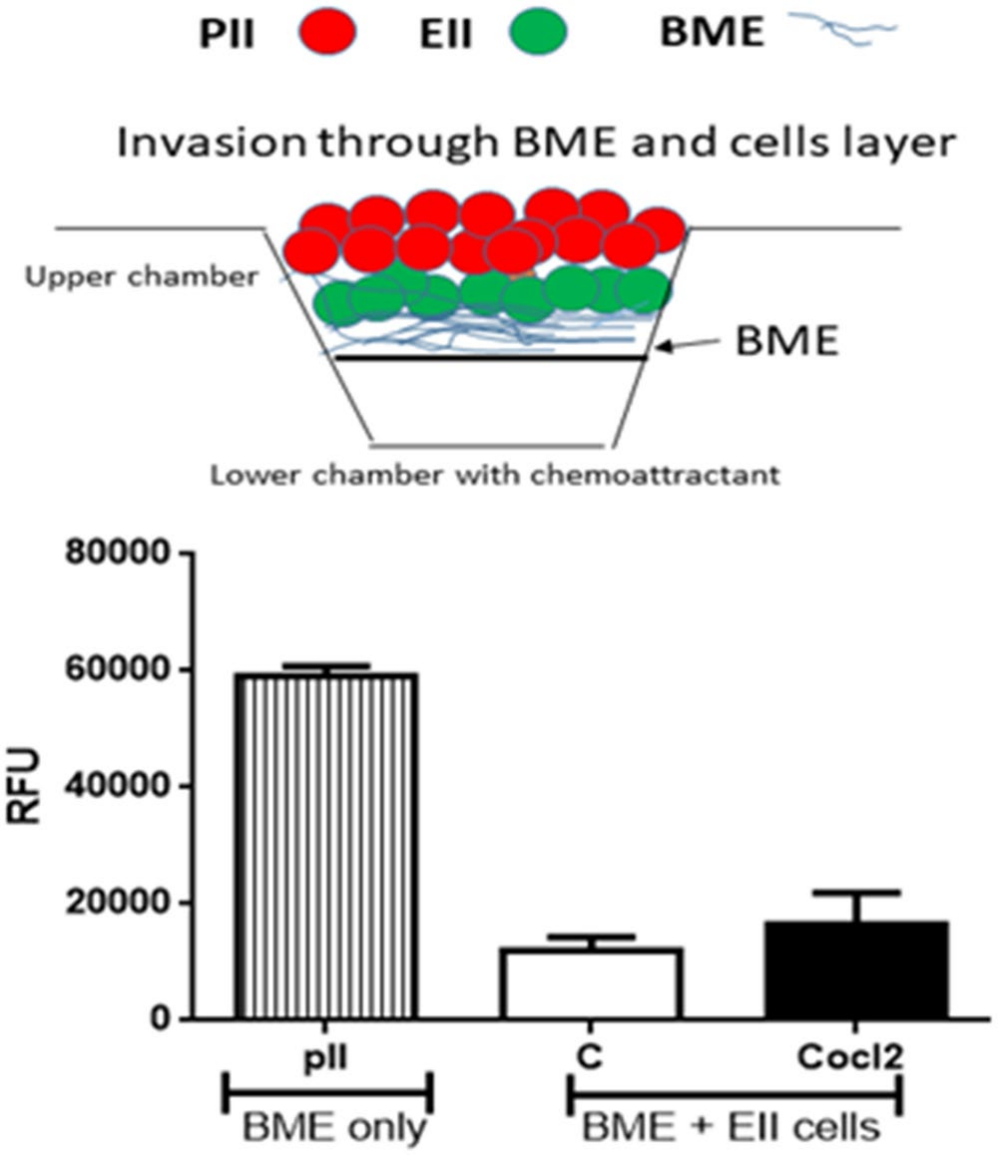
Effect of normoxia and hypoxia on the invasion of ER−ve breast cancer cells through either BME alone or a double layer of ER + cells and BME. Untreated pII cells were loaded into the top chamber of a Cultrex BME cell invasion plate which was previously loaded with BME only. pII cells [treated with vehicle (C) or CoCl_2_, 100 µM] were loaded into the top chamber of a Cultrex BME cell invasion plate which was previously loaded with another layer of EII cells above the BME. pII cells invading into the lower chamber, towards serum, were detected as described in Methods. Histobars represents mean ± SEM of 3 independent determinations. It should be noted that no EII invasion towards the lower chamber was observed. A schematic diagram explaining each experimental setup is shown on the top of the figure.

The Cultrex BME transwell cell invasion system was used to determine whether pII cells could invade through a layer of both EII cells and BME towards serum components. pII cells pre-incubated for 24 h with vehicle (control, normoxia) or with 100 µM CoCl_2_ (hypoxia) were loaded into the top chamber of a transwell plate in which EII cells had previously been seeded on top of a BME layer. After 24 h incubation, only pII cells were found in the lower chamber but the number of these was not significantly different whether pre-exposed to vehicle or CoCl_2._ Moreover, the number of penetrating cells was far fewer than seen in a parallel set-up in which the upper chamber was seeded with a similar number of cells in the absence of EII cells, giving direct contact with the BME layer (Fig. 10). However, it should be noted that the number of invading pII cells through the double barrier of EII/BME were still significantly higher than the number of YS1.2 invading through a single layer of BME (Fig. 7B, upper panel).

## Discussion

An important feature of metastasis is that it appears to involve individual or small groups of cells detaching from the tumor mass and therefore the further question arises – which cells from within the tumor metastasize? Is this a random process or does it involve specific cells? Is it the cells at the tumor periphery or those further inside? Neo-vascularisation of a growing tumor mass is disorganized and incomplete, such that the microenvironment of cells nearer to the core is more hypoxic compared to the cells at the periphery, and this phenomenon may be a major factor in cancer progression and metastasis^6^. It has been reported that hypoxic cells are highly invasive^58,59^, so could these be the cells that metastasize from a tumor? This would entail migration into and possibly through the upper layers of cancer cells that remain adherent, in order to intravasate into physiologically functional blood vessels most likely located at the tumor periphery.

HIF1α is generally over-expressed under hypoxic conditions to regulate expression of downstream genes leading to enhanced cell motility and invasion^60^. HIF1α protein and mRNA levels were elevated in human pancreatic cancer cells in response to (100–200 μM) CoCl_2_ treatment for 0–12 h in a time and dose dependent manner^46^. Also, Chan *et al*.^13^ demonstrated enhanced HIF-1α expression in human lung carcinoma cell lines grown in low O_2_ conditions. DFO treatment (30–300 µM for 24 h) of MDA-MB-231 cells enhanced HIF1α protein expression^61^. In this study, we showed that HIF1α expression in response to CoCl_2_ (100 µM) treatment was significantly increased particularly at 4–24 h of stimulation and declined at 48 h in all the cell lines (Fig. 1A–C). The highest expression (by up to 4-fold) (both at baseline and in response to CoCl_2_) was seen in the ER −ve breast cancer cells (which have a higher proliferation and migration rate in comparison to ER + breast cancer cells), with the lowest expression in the normal epithelial breast cells. DFO treatment of pII cells also enhanced HIF-1α expression to similar levels (Fig. 1D).

Under hypoxic conditions, HIF1α is accumulated into the nucleus^22^. Moroz *et al*.^62^ reported that under normoxic conditions, HIF-1a/FLuc in NIH3T3 reporter cells and HIF-1a in wild-type NIH3T3 cells was mainly localised in the cytoplasm; however, in HEK293 reporter and wild-type cells, HIF1α was distributed between both cytoplasm and nucleus. Upon CoCl_2_ treatment (100 µM, 6 h) HIF1α was translocated to the nucleus in all the cell lines. This pattern was also observed following exposure to hypoxia (2.5% O_2_). In MCF-7 cells, induction of hypoxia using CoCl_2_ treatment (200 µM for 24 h) increased HIF1α nuclear localization and up-regulation of its expression^63^. In agreement with these studies, we too observed cytoplasmic HIF-1α only after hypoxic exposure, with subsequent translocation to the nucleus after 24–48 h.

Hypoxia mediated increase in cell proliferation has been reported in human hepatocellular carcinoma cultured in 1% O_2_ for 36 h^45^ in MCF7 cells treated with CoCl_2_ showing a peak of proliferation at 150 µM with inhibition at higher doses^64^ and similarly in MDA-MB-231 cells, at 25 µM with higher concentrations decreasing proliferation rate^64^. Other reports showed decreased cell growth upon exposure to hypoxic environment. For example, Chan *et al*.^13^ reported that 72 h exposure of human lung carcinoma (H1299) cells to 0.2% O_2_ decreased their survival by 18%, with 0% O_2_ conditions leading to apoptosis. Pancreatic PC2 cancer cells were inhibited by CoCl_2_ (50–200 µM)^46^. Treatment of MDA-MB-231 cells with DFO (30–300 µM, for 24 h) had no effect on their proliferation rate^61^. In this study, we observed that CoCl_2_ and DFO treatment (100 µM, for 24–96 h) decreased both normal and breast cancer cell proliferation (Fig. 5).

Several reports have suggested a role of hypoxia in modulating cancer cell migration and invasion; knockdown of Wnt-1 induced signaling protein 2 (WISP-2) in non-invasive MCF7 cells enhanced their motility under hypoxic conditions (0.2% O_2_, 24 h)^65^ as did treatment of MCF-7 with CoCl_2_ (200 µM, 24 h)^63^ and MDA-MB-231 with DFO (30–300 µM, 24 h). We also demonstrated enhanced ER +ve and ER −ve cell motility (by approximately 2-fold) in response to both CoCl_2_ and DFO treatment (Fig. 6), in agreement with those reports. Hoffmann and colleagues have shown that hypoxia promotes mesenchymal invasion in breast cancer cells mediated through upregulation of cysteine-rich protein 2, a component of the actin cytoskeletal machinery involved in invadopodia formation. This was also demonstrated in xenograft breast tumours *in vivo*^66^.

A somewhat surprising observation was that MCF10A cells were found to be highly motile. However, this was found to be largely due to the presence of EGF in their special culture medium, as they showed little movement when this was omitted. Hypoxic conditions greatly stimulated motility irrespective of the medium used. Maeda and colleagues also observed significant motility (using same medium as ours) that was further enhanced by TGFβ^67^. An interesting aside was their finding that MCF10A exhibited cadherin switching, a phenomenon associated with EMT. Similarly, Rodriguez-Monterrosas and colleagues reported increased motility stimulated by insulin (also a component of the MCF10A medium) in association with EMT^68^. Vaapil and colleagues found no evidence for hypoxia-driven EMT^69^. Such reports have called into question the use of MCF10A as a representative of normal breast epithelia^70^. Or is this a cautionary indication that normal cells can exhibit some ‘cancer-like’ properties confronted with hypoxia or other stimulants? Either way this needs further investigation, but was not pursued here as it is not directly relevant to the objective of this study.

It was noticed that MCF7 cells treated with CoCl_2_ (200 µM for 24 h) migrated faster than control^63^, MDA-MB-231 cell invasion increased by 24–129% by treatment with 30–300 µM of DFO for 24 h^61^ and hepatocellular carcinoma cells treated with 100 µM CoCl_2_ displayed approximately 10% increase in migration and invasion; this effect was reversed upon removal of the CoCl_2_^71^.In agreement with previous observations^56,57^, our data using the Cultrex assay (Fig. 7A,B) shows that ER −ve cells are more invasive through BME when compared to the poorly invasive ER +ve cells but with no further enhancement upon CoCl_2_ or DFO treatment. Random cell migration into agarose, could be seen only with ER- breast cancer cells^56,57^.

To address the issue of whether cells deeper inside a tumor could penetrate through other layers of cells to reach blood vessels, we performed two types of co-culture experiments. We observed that the ER- breast cancer cells were able to make their way into and through a solid barrier of ER + cells when cultured adjacent to each other, and were also able to make their way out from a mixture of cells into the surrounding space (Fig. 8). Hypoxic conditions induced greater number of penetrating cells as compared to the number of invasive cells under normoxic conditions. The number of cells was however very small; possibly due to lack of ‘incentive’ for the cells to move in any particular direction. Although the Ibidi chambers permit visualization of migrating cells they also have disadvantages in this respect. The Cultrex chamber can allow a downward gravitational movement towards a source of serum; using this assay system we observed similar results whereby ER- cells penetrated through a layer of non-invasive ER + cells and invaded through a layer of BME towards serum components. In this assay cell numbers were not counted; their presence was inferred by an indirect measure using calcein uptake. Therefore, we cannot quantitatively compare the two methods but (given that the fluorescence detection method would require a significant number of cells) it would not be unreasonable to assume that much larger numbers got through. Whilst it was observed, as would be expected, that the barrier of EII cells restricted the migration of the pII cells (Fig. 10) it is also clear that some were indeed able to move through the EII barrier. Small increase in number of invasive cells in the presence of CoCl_2_ was not statistically significant. Also, we were able to observe cell penetration through the other cell population only after 3–4 days of co-culture, but in the Cultrex assay, cells need to be assessed within 24 h.

In summary, the present study attempted to construct an *in vitro* model to investigate the possibility that cells that are located deeper within a tumor can penetrate into and migrate through other cell layers in order to reach vascular elements present nearer to the surface of the tumor mass and metastasize. As these are likely to be in a more hypoxic environment (which has been claimed to stimulate metastasis) we performed experiments in both normoxic and hypoxic conditions. Overall our data support the idea of cancer cells metastasizing as an escape from an unfavorable environment, in this case, hypoxia. Certainly, it appears to be a characteristic behavior of cells that have undergone EMT by whatever means Currently there is no data to indicate which cells within a tumor undergo this transformation or where they are located. Intuitively one might expect that it should be the ones at the tumor periphery in close proximity to functional blood vessels; while this may still be the case, our data suggests that EMT transformed cells can also migrate from further inside the tumor. Also, hypoxia significantly enhances endocrine resistant breast cancer cell migration/penetration into other cell monolayers but not through BME. Translating this into an *in vivo* scenario, one might envisage that hypoxia could drive O_2_ deprived cells to migrate towards the peripheral vasculature but not necessarily aid them to intravasate into it.

The biggest limitation of our experimental approach is its two-dimensional nature and the use of pure cell populations. This could be improved with the use of three-dimensional organoid cultures with stromal elements and normal epithelial cells as well as live cell imaging of fluorescently labeled cells to track their movement in real time.

## Supplementary information

Supplementary Figure legends.

Supplementary information 1.

Supplementary information 2.
