# Supplementary material for: Hypoxic environment may enhance migration/penetration of endocrine resistant MCF7- derived breast cancer cells through monolayers of other non-invasive cancer cells in vitro
Source: Sci Rep. 2020 Jan 24;10:1127. doi: 10.1038/s41598-020-58055-x (PMC6981140; doi:10.1038/s41598-020-58055-x)
Supplement: Supplementary file 1 — Supplementary Figure legends. [file 41598_2020_58055_MOESM1_ESM.docx]

**Supplementary legends**

**Supplementary file 1**

**The migrative behavior of endocrine sensitive and resistant breast cancer cells**

A movie of pII (right side) and EII (left side) cells cultured for 48 h. Cells were seeded separately into adjacent chambers of an Ibidi chamber slide with a space between them. After 24 h the scaffold was removed leaving a gap between the two monolayers. Only the pII cells were able to move towards the EII cell monolayer and over 27 h completely closed the gap.

**Supplementary file 2**

**Effect of different culture media and CoCl_2_ or DFO treatment on MCF10A cell motility**

A scratch was made through a confluent layer of MCF10A cells, using a p20 pipette tip, and the gap measured at 0 time and after 24 h of DFO or CoCl_2_ treatment at the concentrations indicated, as described in Methods. The % wound closure was calculated as the width of the empty space after 24 h divided by the width at 0-time x 100 subtracted from 100. Histobars represent the mean ± SEM of 6 independent determinations. Asterisks denote significant difference from the control, with p>0.05 (*), p=0.01 (**), and p=0.001 (***).
