# Supplementary figures and images for: Hypoxic environment may enhance migration/penetration of endocrine resistant MCF7- derived breast cancer cells through monolayers of other non-invasive cancer cells in vitro
Source: Sci Rep. 2020 Jan 24;10:1127. doi: 10.1038/s41598-020-58055-x (PMC6981140; doi:10.1038/s41598-020-58055-x)

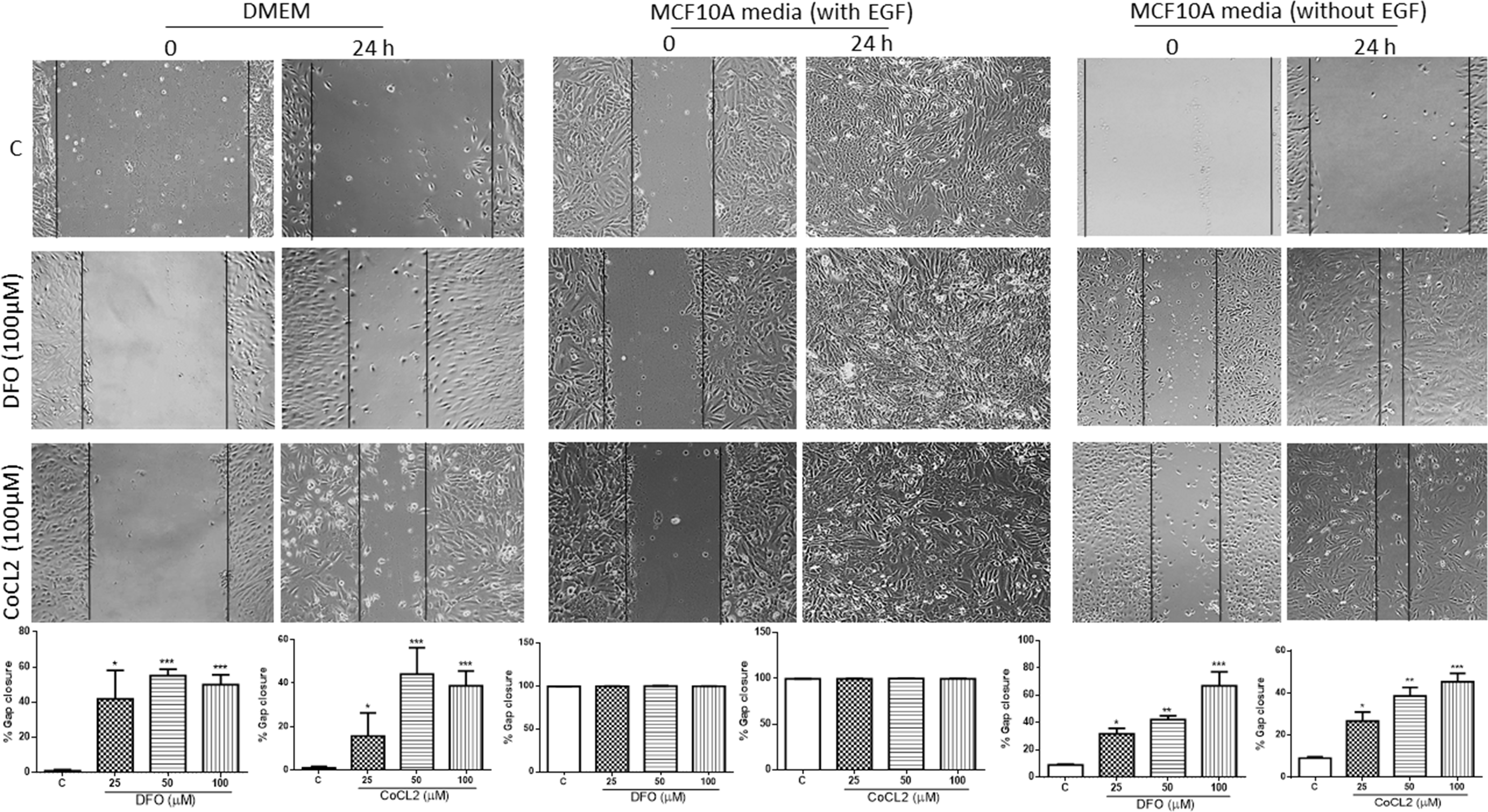

Supplement: Supplementary file 3 — Supplementary information 2. [file 41598_2020_58055_MOESM3_ESM.tif]
